# Interplay between TETs and microRNAs in the adult brain for memory formation

**DOI:** 10.1038/s41598-018-19806-z

**Published:** 2018-01-26

**Authors:** Eloïse A. Kremer, Niharika Gaur, Melissa A. Lee, Olivia Engmann, Johannes Bohacek, Isabelle M. Mansuy

**Affiliations:** 1 Laboratory of Neuroepigenetics, University of Zürich and Swiss Federal Institute of Technology, Brain Research Institute, Neuroscience Center Zürich, Winterthurerstrasse 190, 8057 Zurich, Switzerland; 2 Department of Genetics and Development, Columbia University Medical Center, New York, NY 10032 USA

## Abstract

5-hydroxymethylation (5-hmC) is an epigenetic modification on DNA that results from the conversion of 5-methylcytosine by Ten-Eleven Translocation (TET) proteins. 5-hmC is widely present in the brain and is subjected to dynamic regulation during development and upon neuronal activity. It was recently shown to be involved in memory processes but currently, little is known about how it is controlled in the brain during memory formation. Here, we show that *Tet3* is selectively up-regulated by activity in hippocampal neurons *in vitro*, and after formation of fear memory in the hippocampus. This is accompanied by a decrease in miR-29b expression that, through complementary sequences, regulates the level of *Tet3* by preferential binding to its 3′UTR. We newly reveal that SAM68, a nuclear RNA-binding protein known to regulate splicing, acts upstream of miR-29 by modulating its biogenesis. Together, these findings identify novel players in the adult brain necessary for the regulation of 5-hmC during memory formation.

## Introduction

Epigenetic mechanisms involving DNA methylation are essential for the regulation of gene expression in the brain, and are required for learning and memory formation^1^. Until recently, DNA methylation was believed to be stable in post mitotic cells, but it is now known to be dynamically regulated at specific sites upon neuronal stimulation and learning^2–4^, indicating that it is reversible. While DNA methyltransferases (DNMTs) catalyze DNA methylation on position 5 of cytosines (5-methylcytosine or 5-mC), Ten-Eleven Translocation methylcytosine dioxygenases (TETs) are responsible for DNA demethylation. TET proteins (TET1, 2 and 3) allow demethylation by converting 5-mC into 5-hydroxymethylcytosine (5-hmC)^5^. TETs can further oxidize 5-hmC into 5-formylcytosine and 5-carboxylcytosine that is subsequently excised by the base excision repair pathway^6^. 5-hmC accumulates in the brain during development and is present at high level in the adult brain, suggesting that it likely plays an important role^7^. Like DNA methylation, it is dynamically regulated by neuronal activity^8^ but the mechanisms that allow its dynamic regulation are not known.

TET1 is the best-characterized enzyme among the TET family with regard to learning and memory. *Tet1* mRNA was shown to be downregulated 1 and 3 hours after contextual and cued fear conditioning in area CA1 of the dorsal hippocampus^9^. TET1 regulates the expression of several activity-dependent genes implicated in learning and memory and its overexpression in the hippocampus impairs long-term associative memory^9^. However, global TET1 knockout in mice does not alter memory acquisition and consolidation, but selectively impairs the extinction of hippocampus-sensitive memories^10^. It also affects neurogenesis^11^ and long-term depression in the hippocampus^10^.

Less is known about the role of TET3 in memory processes. However, in the cortex and hippocampus, two brain regions essential for learning and memory, *Tet3* is the most highly expressed enzyme of the TET family^12^. *Tet3* mRNA increases 2 hours after extinction training in the prefrontal cortex, and knockdown of *Tet3* in this region impairs memory extinction, without affecting learning^13^. Thus, TETs might have various roles in memory processes depending on the brain region, and possibly the type of memory.

The modes of regulation of TETs remain unknown but microRNAs (miRNAs) have been thought as potential candidates. MiRNAs are short non-coding RNAs that can control neuronal gene expression required for memory formation. The biogenesis, rapid turnover and combinatorial modes of action of miRNAs make them ideal candidates for a dynamic and reversible regulation of gene expression^14^. They can control multiple targets simultaneously through degradation of their mRNAs or translational repression. Some miRNAs have also been implicated in the regulation of DNA methylation directly by targeting *Dnmts* or indirectly by acting on transcription factors that control *Dnmts* transcription^15,16^. The miR-29 family (a, b and c), in particular, was shown to contribute to epigenetic regulation in cancer by targeting *Dnmt3a* and *b*^16^. Conversely, miRNAs themselves are subject to specific mechanisms of control. Their transcription, processing and degradation are regulated by different processes involving protein-protein and protein-RNA interactions^17^. In these mechanisms, RNA-binding proteins (RBPs) are very important regulators implicated in different stages of miRNAs biogenesis, localization, activity and degradation. SAM68 (also called KHDRBS1), is an RBP mostly known to regulate activity-dependent alternative splicing^18^, was recently shown to influence the expression of a subset of miRNAs in male germ cells^19^. In this study, we provide evidence that the miR-29 family is differentially regulated in the adult hippocampus upon learning and that miR-29 biogenesis is modulated by SAM68. These miRNAs are involved in the control of *Tets*, in particular *Tet3*, which itself is regulated in an activity-dependent manner upon learning.

## Results

### The expression of *Tet* genes is regulated in an activity-dependent manner

**Figure 1:**
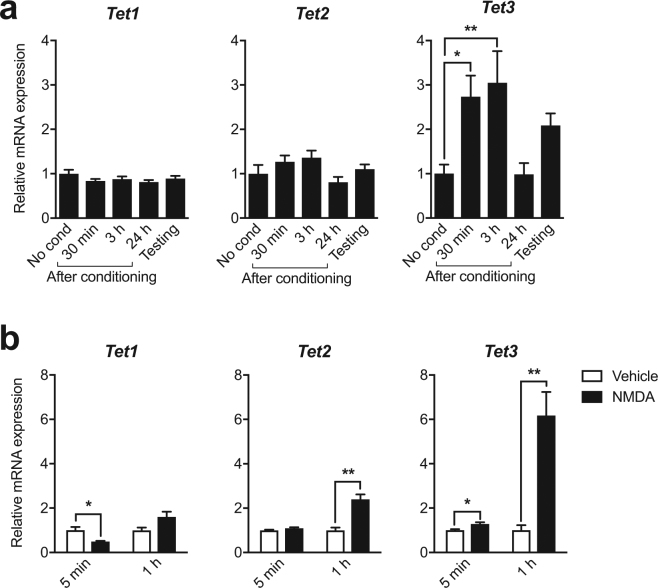
Activity-dependent expression of *Tet* genes. (**a**) Level of *Tet1*, *2*, and *3* in the hippocampus 30 min, 3 h, 24 h after fear conditioning, or 30 min after testing (24 h following conditioning) measured by real-time quantitative reverse transcription-PCR (RT-qPCR). No cond., no conditioning. *p < 0.05, **p < 0.01 determined by one-way ANOVA followed by Dunnett’s post-hoc test (**b**) Level of *Tet1*, *Tet2* and *Tet3* in hippocampal primary neurons 5 min and 1 h after NMDA stimulation (60 μM, 5 min) measured by RT-qPCR. *p < 0.05, **p < 0.01 determined by unpaired t test. Data represent mean ± s.e.m.

To determine the dynamics of TETs regulation upon neuronal activity in the adult brain, we quantified the level of *Tet* mRNAs in the hippocampus after contextual fear conditioning (CFC) (Supplementary Fig. S1). While *Tet1* and *Tet2* mRNA remained unchanged after conditioning, *Tet3* mRNA was up-regulated after 30 min and 3 h but returned to baseline after 24 h (Fig. 1a). To test whether the changes in *Tet3* expression were specific to memory formation in CFC and were not related to the stress response elicited by fear conditioning, we examined the effects of acute cold swim stress on *Tet*s expression in the hippocampus (Supplementary Fig. S2). *C-fos*, an activity-dependent immediate early gene, was used as positive control to ensure that neuronal activation occurred. Importantly, *Tet3* expression was not modified by cold swim stress. Further, *Tet3* expression was also up-regulated by activity in cultured hippocampal neurons *in vitro*. Neuronal activation by NMDA, confirmed by *C-fos* expression (Supplementary Fig. S3a), significantly increased *Tet3* expression after 5 min and 1 h (Fig. 1b). It also slightly increased *Tet2* expression after 1 h but decreased *Tet1* after 5 min, suggesting a dissociated response of the three TETs. Consistently, *C-fos, Tet2* and *Tet3* but not *Tet1* were also up-regulated by activation of NMDA receptors by the co-agonist glycine *in vitro* (Supplementary Fig. S3b,c). These findings suggest that NMDA receptor signaling increases *Tet3* transcription, both after neuronal activation *in vitro* and learning *in vivo*.

### The expression of the miR-29 family is regulated in an activity-dependent manner

**Figure 2:**
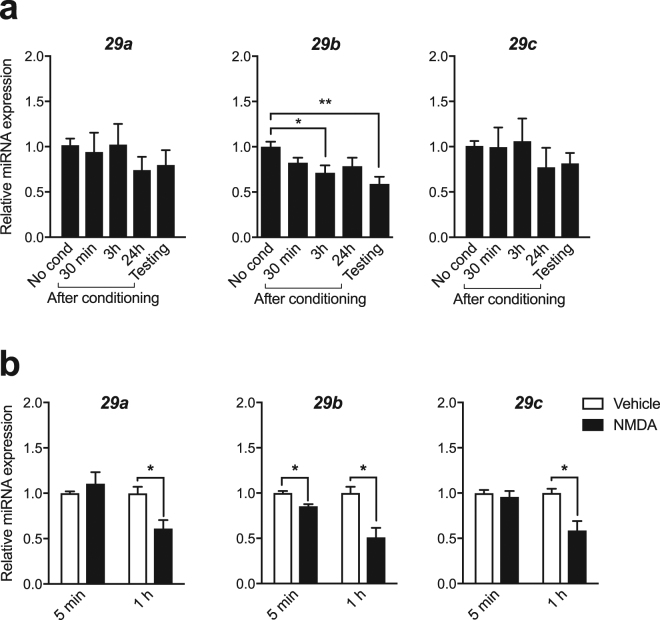
Activity-dependent expression of miR-29 family. (**a**) Level of miR-29a, b and c in the hippocampus 30 min, 3 h, 24 h after fear conditioning, or 30 min after testing (24 h after conditioning) measured by RT-qPCR. *p < 0.05, **p < 0.01 determined by one-way ANOVA followed by Dunnett’s post-hoc test. No cond., no conditioning; (**b**) Level of miR-29a, b and c in hippocampal primary neurons 5 min and 1 h after NMDA stimulation (60 μM, 5 min) measured by RT-qPCR. *p < 0.05 determined by unpaired t test. Data represent mean ± s.e.m.

We next sought to identify which mechanisms participate to the control of *Tet3* mRNA level. I*n silico* target gene prediction algorithms indicated that *Tet* 3′-UTR has multiple well-conserved binding sites for miR-29 (Supplementary Table S1). Therefore, we examined whether miR-29, a miR cluster including miR-29a, b and c, has a relation to *Tets* during CFC. While miR-29a and c remained constant, miR-29b expression was significantly down-regulated 3 h after conditioning and following testing (Fig. 2a). MiR-29b was also decreased 5 min and 1 h after NMDA stimulation in hippocampal neurons, while miR-29a and c expression decreased only after 1 h (Fig. 2b). Likewise, it was decreased after 5 min and 1 h of glycine treatment while miR-29a and c were not (Supplementary Fig. S4). These results suggest that miR-29b expression is activity-dependent and has an expression profile inverse to *Tet3* after learning and neuronal activity.

### **MiR-29b modulates the expression level of*Tet*genes, in particular*Tet3***

**Figure 3:**
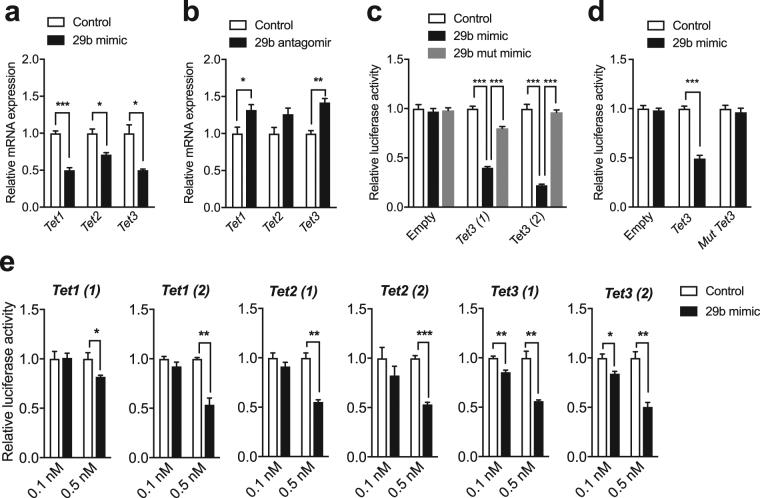
MiR-29b preferentially controls *Tet3* expression level. (**a**) Level of *Tet1*, *Tet2*, and *Tet3* in N2a cells after transfection with miR-29b mimic or control measured by RT-qPCR. *p < 0.05, ***p < 0.001 determined by unpaired t test. (**b**) Level of *Tet1*, *Tet2*, and *Tet3* in N2a cells after transfection with miR-29b antagomir or control measured by RT-qPCR. *p < 0.05, **p < 0.01 determined by unpaired t test. (**c**) Analysis of *Tet3* luciferase reporters in the presence of miR-29b mimic, seed mutant miR-29b mimic or control (40 nM) in N2a cells. ***p < 0.001 determined by one-way ANOVA followed by Tukey’s post-hoc test. (**d**) Analysis of *Tet3* 3′UTR luciferase reporter and seed sequence mutated *Tet3* 3′UTR luciferase reporter in the presence of miR-29b mimic. ***p < 0.001 determined by unpaired t test. (**e**) Analysis of *Tet1, Tet2* and *Tet3* luciferase reporters in the presence of graded concentrations of miR-29b mimic or control in N2a cells. *p < 0.05, **p < 0.01, ***p < 0.001 determined by unpaired t test. Data represent mean ± s.e.m.

To test if miR-29b targets *Tets*, we manipulated its level in N2a cells using miRNA mimic or antagomir. Overexpression of a miR-29b mimic down-regulated *Tet1, 2* and 3 expression (Fig. 3a) while a seed-mutant miR-29b mimic had no effect (Supplementary Fig. S5). In addition, miR-29b knockdown increased *Tet1* and 3 expression (Fig. 3b). Then, using two different regions of *Tet3* 3′-UTR (*Tet3*(1) and *Tet3*(2)) with seed sequences for miR-29 cluster (Supplementary Fig. S6a) and a luciferase reporter, we further examined the interaction between *Tet3* and miR-29b. MiR-29b mimic significantly reduced luciferase activity with both reporters (Fig. 3c), showing that miR-29b regulates the level of *Tet3* mRNA likely by binding to its 3′UTR. However, a seed-mutant form of miR-29b mimic had no or minimal effect on luciferase activity of *Tet3* reporters (Fig. 3c). Similarly, seed mutation of miR-29 cluster within *Tet3* 3′UTR did not affect luciferase activity compared to its non-mutated form (Fig. 3d).

Additionally, high doses of miR-29b mimic reduced the luciferase activity of *Tet1* and *Tet*2 reporters (Supplementary Fig. S6b). To determine whether miR29b preferentially regulates *Tet3*, we tested the effect of graded concentrations of miR-29b mimic. While overexpression of miR-29b by mimic had no effect on the control vector (Supplementary Fig. S7), it decreased the expression of *Tet3* luciferase reporters in a dose-dependent manner. At low concentration (0.1 nM), the miR-29b mimic repressed *Tet3* luciferase reporters but had no effect on *Tet1* and *Tet*2, indicating a dose-dependent target regulation of miR-29b (Fig. 3e). Using STarMiR^20^ to model mRNA secondary structure, we found that *Tet*3 3′UTR contains more accessible miR-29b binding sites than *Tet1* and *Tet*2 3′UTRs (Supplementary Table S2), providing a potential explanation for our experimental findings. As expected, miR-29b overexpression decreased *Dnmt*3*a* and *b* mRNA level as well as the luciferase activity of a *Dnmt*3*a* reporter (Supplementary Fig. S8). However, *Dnmt1* was not changed, consistent with the absence of binding sites for miR-29s in its 3′-UTR (Supplementary Fig. S8a), confirming the specificity of the assay.

### **Synaptic and memory-related genes are sensitive to changes in*Tet3*levels**

**Figure 4:**
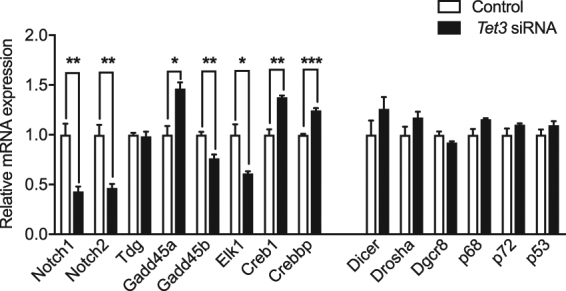
TET3 regulates the expression of synaptic and memory-related genes. Transcriptional analysis of genes involved in synaptic plasticity, memory formation, and miRNA biogenesis after *Tet3* knockdown in N2a cells measured by RT-qPCR. *p < 0.05, **p < 0.01, ***p < 0.001 determined by unpaired t test. Data represent mean ± s.e.m.

To further investigate the function of TET3, we examined the effects of *Tet3* knockdown on the expression of potential candidate genes. Using a pool of siRNAs specifically targeting *Tet3* (*Tet1* and *2* remained unchanged, Supplementary Fig. S9), we identified genes involved in Notch signaling (*Notch1* and *2*), repair-based DNA demethylation (*Gadd45a* and *b)* and transcriptional activation (*Elk1, Crebbp* and *Creb1*) (Fig. 4). To control for potential off-target effects, we also used a single siRNA targeting *Tet3* and could replicate our findings (Supplementary Fig. S10). However, *Gadd45b* expression was not altered upon transfection of a single *Tet3* siRNA, suggesting that this candidate may result from an off-target effect. Interestingly, all these genes were previously implicated in neuronal activity, synaptic plasticity and/or memory processes^21–23^. Therefore, we further examined their expression in the hippocampus following CFC. The expression of *Notch1*, *Creb1*, *Crebbp* and *Gadd45b* was found to be significantly altered 30 min after training, a time-point at which *Tet3* expression is high (Supplementary Fig. 11). This supports a role for TET3 in the modulation of these genes upon learning.

In contrast, the expression of major components of the miRNA biogenesis *(Drosha*, *Dgcr8*, *Dicer)* and other microprocessor accessory proteins involved in the control of miRNA biogenesis such as *p68*, *p72* and *p53* were not altered by *Tet3* knockdown (Fig. 4), indicating that TET3 does not affect global miRNA biogenesis. These results suggest that an intermediate molecular player is involved in modulating the expression of miR-29b upon neuronal activity.

### SAM68 is involved in the transcriptional regulation of miR-29s

**Figure 5:**
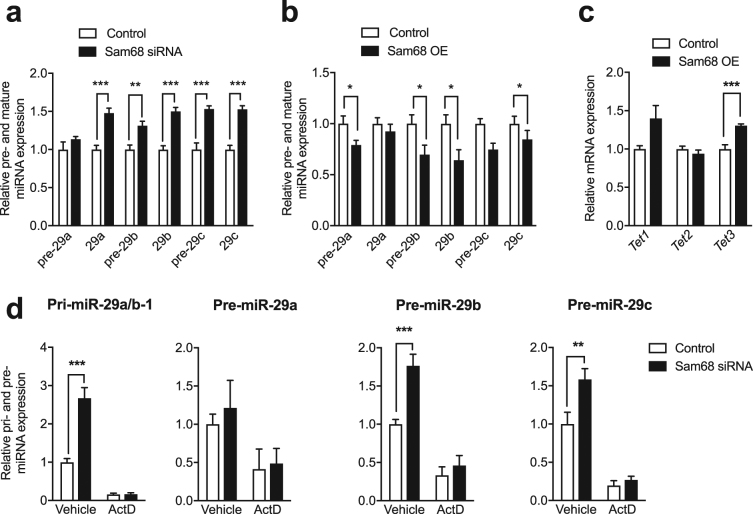
SAM68 modulates the biogenesis of miR-29s at the transcriptional level. (**a**) Level of precursor and mature miR-29a, b and c in N2a cells after *Sam68* knockdown. **p < 0.01, ***p < 0.001 determined by unpaired t test (**b**) Level of precursor and mature miR-29a, b and c in N2a cells after *Sam68* overexpression. *p < 0.05 determined by unpaired t test. (**c**) Level of *Tet1, 2* and *3* after overexpression of *Sam68* or control measured by RT-qPCR. ***p < 0.001 determined by unpaired t test. (**d**) Level of nuclear pri-miR29a/b-1, pre-miR-29a, pre-miR-29b and pre-miR-29c transcripts in N2a cells after *Sam68* knockdown measured by RT-qPCR in the presence of ActD treatment or vehicle. **p < 0.01, ***p < 0.001 determined by two-way ANOVA followed by Bonferroni’s post-hoc test. Data represent mean ± s.e.m.

Previous work has identified that SAM68 - an RBP - influenced the expression of specific miRNAs in male germ cells, including miR-29b^19^. RBPs play a role in the biogenesis of specific miRNAs (reviewed in^24^), thus we examined the potential link between SAM68 and its role in regulating the biogenesis of the miR-29 family. MiRNAs are produced through the action of multiple enzymatic steps involving the transcription of primary miRNAs (pri-miRNAs), their processing into precursors miRNAs (pre-miRNAs) in the nucleus and then into mature miRNAs in the cytoplasm^14^. To determine whether miR-29 biogenesis is modulated by SAM68, we quantified the level of pre- and mature miR-29s after *Sam68* knockdown. We found that pre-miR-29b and pre-miR-29c were significantly up-regulated in N2a cells (Fig. 5a). Similarly, *Sam68* knockdown (Supplementary Fig. S12a) led to an increase in mature miR-29a, b and c (Fig. 5a). Although previous studies found that miR-182 and miR-10b are dynamically regulated after fear conditioning^25,26^, the expression of these miRNAs was not affected by *Sam68* knockdown, showing a clear selectivity of the effect (Supplementary Fig. S12b). Conversely, overexpression of *Sam68* resulted in a significant decrease in precursor and mature miR-29b, confirming that SAM68 is implicated in the biogenesis of these miRNAs (Fig. 5b). Interestingly, we also found significant changes in *Tet3* expression level, suggesting that both SAM68 and miR-29b are involved in the regulation of *Tet3* (Fig. 5c).

Because *Sam68* modulation alters both precursor and mature forms of miR-29b, it indicates that SAM68 likely exerts its regulatory effect at the transcriptional level. To further investigate the mechanisms by which SAM68 modulates miR-29 biogenesis, we used the transcription inhibitor actinomycin D (ActD) to test whether transcription is responsible for the up-regulation of precursors and mature forms of miR-29b. While *Sam68* knockdown led to an increased level of pri-miR-29a/b-1 transcripts, as well as pre-miR-29b and pre-miR-29c, ActD treatment blocked these changes (Fig. 5d). This suggests that SAM68 likely acts upstream of RNA Pol II-dependent transcription to regulate miR-29b. To determine whether the expression of *Sam68* is regulated by activity, we quantified the level of *Sam68* transcripts in hippocampal neurons after NMDA stimulation. *Sam68* was significantly increased 5 min after neuronal activity, but not 1 h later (Supplementary Fig. S13). Therefore, we propose that transient increase in *Sam68* inhibits pri-miR-29a/b transcription, thereby reducing mature miR-29b levels thus reinforcing *Tet3* transcriptional program.

## Discussion

The present data demonstrate that *Tet3* expression is preferentially increased in the hippocampus after learning. This effect is, however, transient, as *Tet3* mRNA levels come back to baseline 24 h after training. Neuronal stimulation through activation of NMDA receptors in primary hippocampal neurons increases *Tet3* mRNA levels, indicating NMDA-dependent regulation of *Tet3* expression. These results are consistent with previous data showing regulation of *Tet3* expression upon global synaptic activity changes^27^. Thus, *Tet3* activity-dependence may explain its up-regulation in the hippocampus after learning. Although *Tet2* expression is not altered in the hippocampus after training, it is significantly up-regulated upon NMDA stimulation in primary neurons, suggesting that *Tet2* is also activity-dependent. It is possible that this up-regulation is neuron-specific, but may also affect glial cells. In agreement with previous reports, *Tet1* transcripts were found to decrease *in vitro*^9^. Although Kaas *et al*. confirmed the effect *ex vivo* using the CA1 subregion of the hippocampus, we did not observe any significant change in the whole hippocampus in adult mice. Even though all TETs share the same enzymatic activity, TETs seem to be differentially recruited in specific sub-regions of the hippocampus upon learning, and thus, may contribute to different biological processes. Following acute stress, the level of *Tet3* transcripts in the hippocampus was not changed, indicating that *Tet3* is specifically regulated upon learning but not stress. We further sought to determine whether the level of 5-hmC resulting from TETs activity, was altered in the brain upon learning. In this study, however, we did not detect any change in global level of 5-hmC in the hippocampus following CFC using ELISA (Supplementary Fig. S14), likely due to the low sensitivity of the method.

The change in *Tet3* expression level after CFC inversely correlates with that of its targeting miRNA, miR-29b. Stimulation of NMDA receptors in cultured hippocampal neurons also lead to alterations in miR-29 expression levels, indicating that changes in miR-29s, similarly to *Tets*, occur in an NMDA dependent-manner. Changes in miR-29b expression likely orchestrate a temporal regulation of *Tet* expression associated with learning by either releasing its mRNAs from miRNA-mediated destabilization, and/or acting as a fine-tuner of gene expression by reinforcing *Tet3* transcriptional program. Several miRNAs have been previously reported to exhibit dynamic expression following neuronal activity^25^, suggesting that coordinated changes in miRNA expression contribute to the regulation of newly synthetized activity-dependent mRNA targets. Specifically, neuronal activity was found to decrease the expression of most neuronal miRNAs in the hippocampus^28^. This is in line with a regulatory network in which miRNAs maintain transcripts in a repressed state until relieved by neuronal activity.

Threshold response in target gene expression by miRNAs has been proposed as a mode of gene regulation by miRNAs^29^. Therefore, if the pool of *Tet1, 2* and *3* mRNA is below the saturation regime of miR-29b, then all *Tets* will be similarly repressed regardless of expression level. However, as *Tet3* mRNA level raises following neuronal activity, target de-repression due to miR-29b saturation might occur. As *Tet1* mRNA level drops upon neuronal activity, *Tet1* is likely to be subjected to constant repression. Interestingly, a study reported that gene transcripts up-regulated after CFC have more predicted miRNA binding sites in their 3′-UTR than down-regulated ones^25^, suggesting that down-regulated transcripts are less likely to be regulated by miRNAs. In agreement with this observation, *Tet1* 3′-UTR has fewer putative miR-29 binding sites than *Tet3* 3′-UTR even if longer (Supplementary Table S1).

Although miR-29b regulates mRNA levels of all members of the TET family through complementary binding to their 3′UTRs, we found that a low amount of miR-29b preferentially regulates *Tet3* and to a lesser extent *Tet2* while a higher amount affects all *Tets*. The extent of target gene repression by miRNAs depends on the expression level of individual miRNAs, as well as, their targets. In support of this idea, TET1 displays low expression in the adult brain, while TET2 and 3 are relatively abundant, in particular in the hippocampus and cortex^12^. Additionally, miR-29a and b have a relatively broad expression pattern in the brain^30,31^. The number, position, and co-operation of miRNA binding sites within the 3′UTR, and the secondary structures of target mRNAs are additional relevant factors that determine the strength of miRNA-mediated gene repression^32–35^. Accordingly, the predicted secondary mRNA structure of *Tet3* contains more accessible binding sites for miR-29b than *Tet1* and *Tet2* (Supplementary Table S2), suggesting that *Tet3* is more likely to be under the regulation of miR-29b. Consistently, *Tet3* mRNA in the hippocampus was found to be highly enriched in the fraction of AGO2-bound mRNAs while *Tet1* could not be detected, suggesting that in the brain *Tet3* is more likely to be a target of miRNAs^36^. However, this also suggests that miR-29b might not be the only candidate in the upstream regulation of *Tet3* expression.

The biogenesis of miRNAs is extensively controlled by protein interactors to ensure cell/tissue specific functions or appropriate response to stimuli. One of the largest groups of proteins that has been recognized as important modulator of miRNA biogenesis and function are RBPs. We provide evidence that the RBP SAM68 is involved in the biogenesis of the miR-29 family. *Sam68* knockdown leads to increased expression of pri-miR-29a/b, precursor and mature miR-29b, while *Sam68* overexpression induces a decrease in the expression of precursor and mature miR-29b. This suggests that SAM68 is likely to mediate its negative regulatory effect at the transcriptional level. Consistently, SAM68 was previously shown to play a role in transcriptional regulation that is independent of its RNA binding activity^37,38^. We further found that *Sam68* expression is transiently regulated by neuronal activity in hippocampal neurons. Previous work has shown that neuronal activity triggers activation of SAM68 through phosphorylation at serine 20^18^, providing evidence that SAM68 is controlled in an activity-dependent manner. Consequently, SAM68 is likely to modulate miR-29b transcription upon neuronal activity, ultimately leading to reduced mature miR-29 levels, thus allowing *Tet3* transcripts to increase.

Activity-dependent increase of *Tet3* in the hippocampus after CFC is proposed to impact on the transcriptional activity of genes related to contextual memory formation. In line with this hypothesis, TET3 was recently identified as a critical regulator of activity-induced gene expression in cultured neurons. In this study, a high proportion of genes, which expression changes upon neuronal activity, lost responsiveness after *Tet3* knockdown^27^. Our transcriptional analyses revealed that synaptic plasticity and memory-related genes are sensitive to changes in TET3 levels. Among the transcriptional target of TET3, we identified the transcription factors CREB1 and ELK1, as well as the coactivator CREBBP, which are known to play a pivotal role in the formation of long-term memory^39^ via the regulation of immediate early genes such as C-*fos*. Other TET3-sensitive loci include genes involved in active DNA demethylation such as *Gadd45a* and possibly *Gadd45b*. Consistently, Kaas *et al*. identified genes encoding enzymes that act downstream of TET-mediated 5-mC oxidation, including *Tdg*, *Apobec1*, *Smug1* and *Mbd4*, to be sensitive to TET1 protein levels. In addition, mapping of TET3 genomic binding sites in the embryonic mouse brain revealed TET3 selective targeting of base excision repair genes^40^. We further demonstrate that *Notch1* and Notch2 expression levels decrease upon *Tet3* knockdown. Interestingly, previous work has shown that Notch signaling is induced in neurons by increased activity, and conditional knockout of *Notch1* in the hippocampus alters synaptic plasticity and memory acquisition^41^. In addition, many genes encoding NOTCH signaling components were previously identified to present activity-induced CpG (de)methylation and expression changes in response to neuronal stimulation in the dentate gyrus^2^. TET3 may therefore contribute to the epigenetic control of genes involved in NOTCH signaling pathway upon neuronal activity. Importantly, we also demonstrate that most of these genes are changed following CFC, supporting the idea that TET3 regulates their expression.

In neuronal cells, TET3 binding was demonstrated to be targeted to genes involved in mRNA processing and splicing, including *Sam68*^40^. This observation raises the intriguing possibility that *Sam68* is sensitive to TET3 levels. We indeed found that *Sam68* is up-regulated upon *Tet3* knockdown, while its expression is reduced upon *Tet3* overexpression in N2a cells (Supplementary Fig. S15). Based on these findings, we propose that increased TET3 levels negatively affect *Sam68* gene expression, and this regulatory loop allows for TET3 transient expression upon neuronal activity (Supplementary Fig. S16). Indeed, *Sam68* expression is regulated oppositely to *Tet3* expression (Supplementary Fig. S17).

Although specific genes implicated in learning and memory were demonstrated to be susceptible to TET3-mediated transcriptional regulation, little is known about TET3 involvement in memory processes. The only demonstration that TET3 may contribute to memory processes comes from a study by Li *et al*., which reported that *Tet3* knockdown in the prefrontal cortex is associated with impaired extinction learning^13^. Further investigations will be required to determine what are the effects of TET3 depletion or overexpression in the hippocampus on memory performance. Furthermore, the effects of miR-29 depletion on 5-mC and 5-hmC profiles and the impact on learning and memory formation remain unknown. Importantly, miR-29a and b have previously been shown to affect synapse formation and plasticity^42^ and have been linked to neurodegenerative disease such as Alzheimer’s^30^. As DNA (de-)methylation is essential for memory formation and plasticity, disrupting the SAM68-miR-29s-TETs regulatory circuit may interfere with physiological functions and contribute to the etiology of neurodegenerative disorders.

## Methods and Materials

### Animals

C57Bl/6 J mice were maintained under a reverse light-dark cycle in a temperature and humidity-controlled facility with food and water *ad libitum*. All experimental manipulations used in this study were approved by the cantonal veterinary office of Zurich and performed in accordance with the relevant guidelines and regulations. All behavioral tests were conducted in adult male animals by experimenters blind to treatment.

### Contextual fear conditioning

Mice were handled for three days prior to training and testing. Mice were trained in a contextual fear conditioning (CFC) paradigm (TSE). They were placed in the chamber (context) for 2 mins before receiving three brief electric foot-shocks 1 min apart (0.3 mA for 1 s) followed by another 2 min in the chamber. Fear conditioned animals were euthanized 30 min, 3 hours, 24 hours after conditioning. Control animals were exposed to the same chamber for the same duration but received no foot shock and were sacrificed 30 min later. Mice were tested 24 hours after training by re-exposure to the context in the absence of foot-shock. Freezing response was measured for 2 min immediately before and 24 h after fear conditioning and was reported as a percentage of time.

### Forced swim test

Mice were placed in a small tank of water (18 cm high, 13 cm diameter, 18 ± 1 °C, filled up to 12 cm) for 6 min. Floating duration was scored manually.

### Brain tissue collection and processing

Immediately after sacrifice, the brain was removed and the hippocampus rapidly dissected on ice and stored at −80 °C. To avoid potential hemispheric lateralization, both hippocampi were pooled and cryohomogenized as previously described^43^.

### Cell culture

Mouse neuroblastoma (N2a) cells (from ATCC) were cultured in Dulbecco’s modified eagle medium (DMEM-high glucose) supplemented with 10% (v/v) fetal bovine serum (Gibco^®^) and 1% Antibiotic-Antimycotic (Gibco^®^). Cells were treated with 40 nM miScript miRNA mimic or inhibitor (Qiagen) and a negative control siRNA with no known target in mammalian genomes (All Stars Negative siRNA, Qiagen). Transfections were carried out using lipid-based HiPerfect transfection reagent (Qiagen). Cells were harvested 24 h after transfection by removing the medium, washing with PBS, and total RNA was isolated using standardized Trizol protocol. Transfection with single or a pool of siRNAs directed against *Tet3* or *Khdrbs1* (Flexitube Gene Solution, Qiagen) and negative control siRNA (All Stars Negative siRNA, Qiagen) was carried out with Lipofectamine^®^ 2000 transfection reagent (ThermoFischer Scientific) according to the manufacturer’s recommendations. In actinomycin D (Tocris®) treatment conditions, the cells were treated with 2.5 µg/ml of the drug prepared in DMSO for 2 h before harvest. Overexpression of *Tet3* was performed using Purefection reagent (System Bioscience) according to the manufacturer’s recommendations. Plasmid pEF-DEST51 containing *Tet3* ORF with the CxxC DNA-binding domain was a kind gift from Prof Gerd Pfeifer. The pEGFP-C1-SAM68 plasmid (kindly provided by Prof Peter Scheiffele) was used for overexpression of *Sam68* in combination with Lipofectamine^®^ 2000 according to the manufacturer’s recommendations.

### Primary neuronal culture

Neuronal hippocampal cultures were prepared from E-18 embryos and grown in Neurobasal medium supplemented with B27, 1 µg/µl gentamycin, 2 mM glutamax. NMDA stimulation was induced by incubating neurons (11 DIV) for 5 min with 50 µM NMDA, after which neurons were returned to fresh medium. Glycine stimulation was induced in 11 DIV hippocampal cultures as previously described^44^. Briefly, activation of NMDA receptors was achieved by incubating neurons for 3 min with saturating levels (200 μM) of the co-agonist glycine, in Mg^2+^-free extracellular medium. Neurons were harvested 5 min or 1 h after stimulation in Trizol® reagent (Invitrogen).

### miRNA targets prediction

TargetScan6.2^45^, which is based on potential binding site in the 3′ untranslated region of the mRNA and predicted stable thermodynamic binding, was used to predict miRNAs that target *Tet*s. Secondary structures of miR-29s binding sites to *Tet3* 3′-UTR and mimimum free energy were predicted according to RNAfold^46^ or STarMir^20^.

### RNA extraction and real-time quantitative reverse transcription-PCR (RT-qPCR)

Mouse hippocampal tissue was homogenized using TissueLyser (Qiagen) in Trizol® reagent (Invitrogen). Total RNA was isolated according to the manufacturer’s recommendations. Subcellular fractionation of nuclear and cytoplasmic RNA was performed using Norgen’s Cytoplasmic and nuclear RNA purification kit (Norgen BioTek Corp). Nuclear RNA was further treated with RNase-free DNase I kit (Norgen BioTek Corp) to remove genomic DNA contaminations. For mRNAs, total RNA was reverse-transcribed using M-MLV reverse transcriptase (Promega). Data for brain samples were normalized to two endogenous controls *Gapdh* and *Actb*, and data for cellular samples were normalized to *Tubd1* and *Hprt1*. Cycling conditions: 5 min at 95 °C, 45 cycles with denaturation (10 s at 95 °C), annealing (10 s at 60 °C) and elongation (8–10 s at 72 °C). For miRNAs, total RNA was reverse-transcribed using miScript II reverse transcription kit^®^ (Qiagen). MiScript primer assays for mature and precursor miRNAs (Qiagen) were used to amplify the respective transcripts from a cDNA pool. RT-qPCRs were performed in a LightCycler 480 qPCR (Roche) using SYBR Green (Roche) according to the manufacturer’s recommendations. Ribosomal *Rnu6* and *Snord61* were used for normalization of Ct values for miRNAs. The primer sequences used for the quantification of mRNAs and miRNAs are shown in Supplementary Table S3.

### Luciferase reporter assays

For validation of *Tet1, 2, 3* and *Dnmt3a* targeting by miR-29b, segments of their 3′UTR including miR-29b seed sequences were amplified from mouse genomic DNA and cloned into pmirGLO Dual-Luciferase miRNA target expression vector (Promega). For miRNA seed mutagenesis of *Tet3* 3′UTR, mutations were predicted by ImiRP^47^, a mutation generator program that enables selective disruption of miRNA target sites while ensuring predicted target sites for other miRNAs are not created. *Tet3* 3′UTR containing mutated seed sequences was synthesized by IDT and cloned into pmirGLO.

N2a cells were co-transfected with miR-29b-3p mimic (5′-U**AGCACCA**UUUGAAAUCAGUGUU-3′ from Qiagen), seed sequence mutated miR-29b-3p mimic (5′-U**CAGCAAC**UUUGAAAUCAGUGUU-3′ from Qiagen) or negative control (All Stars Negative siRNA, Qiagen) and 250 ng of pmirGLO with Lipofectamine^®^ 2000 (Life Technologies) for 24 h. Cell extracts were prepared 24 h post-transfection, and luciferase activities of firefly and renilla were measured using a Dual-Luciferase Reporter Assay system (Promega) and a luminometer GloMax 96 (Promega). Firefly luciferase signals were normalized to Renilla luciferase signals, which serve as internal normalization control. Values were further normalized by that of an empty pmirGLO vector. The primer sequences used for cloning are shown in Supplementary Table S4.

### Global 5-hmC quantification by ELISA

Genomic DNA from mouse hippocampal tissue was isolated using AllPrep DNA/RNA/miRNA Universal kit (Qiagen). Quantification of 5-hmC was determined using MethylFlash Hydroxymethylated DNA 5-hmC Quantification Kit (Colorimetric) (EpiGentek), according to the manufacturer’s instructions.

### Statistical analysis

Statistical comparisons between two groups were performed using an unpaired Student *t*-test. One or two-way ANOVA were performed followed by Dunnett’s, Tukey’s and Bonferroni’s *posthoc* analyses when appropriate. All analyzed data matched the requirements for parametric statistical tests (normal distribution). If variance was not homogenous between groups (determined by Browth-Forsythe’s test), adjusted *P* value, *t* value and degree of freedom were determined (Welch correction). Values over two standard deviations away from the mean of each group were considered outliers and excluded from analysis. All statistics were computed with Graphpad Prism. All reported replicates were biological replicates. Significance was set at *P* < 0.05 for all tests. Error bars represent s.e.m. in all figures.

### Data availability

All data generated or analysed during this study are included in Supplementary Table S5.

## Electronic supplementary material

Supporting information

Supplementary Table S5
