# Supplementary material for: Interplay between TETs and microRNAs in the adult brain for memory formation
Source: Sci Rep. 2018 Jan 26;8:1678. doi: 10.1038/s41598-018-19806-z (PMC5786039; doi:10.1038/s41598-018-19806-z)
Supplement: Supplementary file 1 — Supporting information [file 41598_2018_19806_MOESM1_ESM.pdf]

Revised manuscript

Supporting Information

Interplay between TETs and microRNAs in the adult brain for memory formation

Eloïse A. Kremer, Niharika Gaur, Melissa A. Lee, Olivia Engmann, Johannes Bohacek, and Isabelle M. Mansuy

SUPPLEMENTARY FIGURES

Supplementary Figure S1

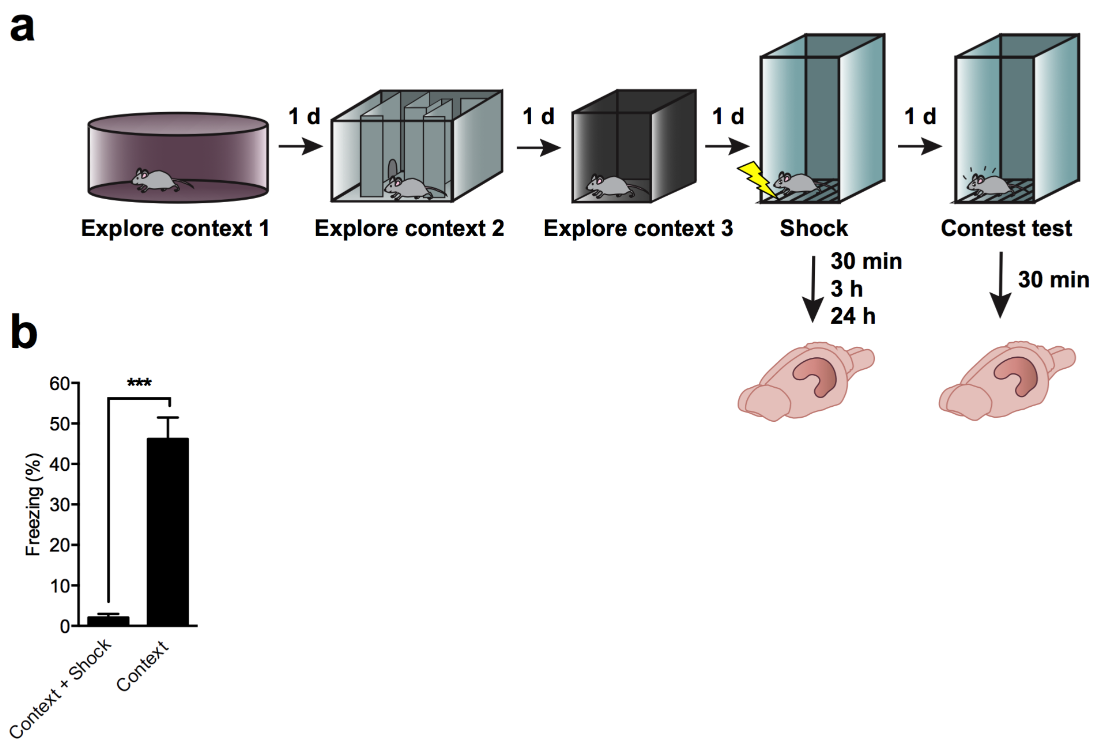

Supplementary Fig. S1. Contextual fear conditioning paradigm

(**a**) During habituation, a mouse was familiarized to an environment different from the home cage (context 1, 2 and 3) and was then placed in a novel context, where it receives 3 electric footshocks. After 24h, the animal was placed back in the context without any shock. The freezing response was measured as an indicator of fear memory. (**b**) Left bar shows baseline freezing before delivery of the foot shock, right bar shows freezing during context test (24h after conditioning). ***p≤0.001 determined by unpaired t test. Data represent mean ± s.e.m.

Supplementary Figure S2

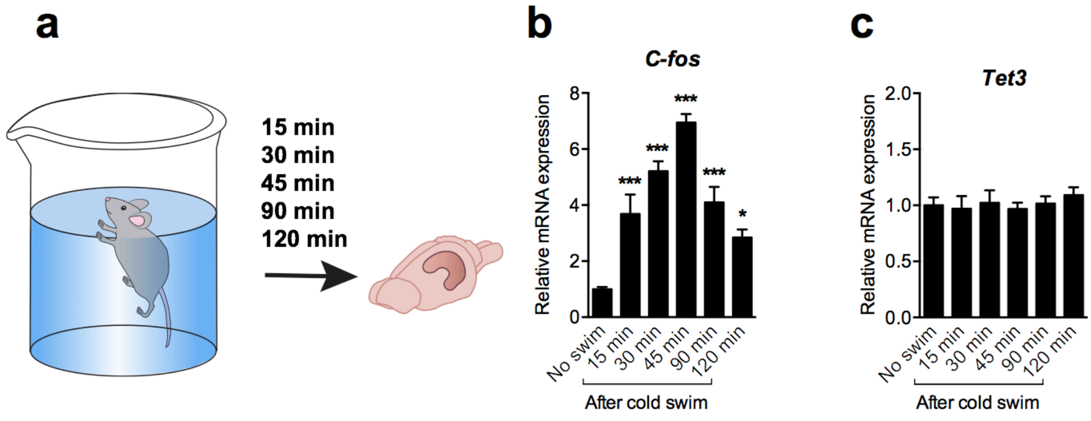

Supplementary Fig. S2. *Tet3* is not responsive to stress

(**a**) Mice were placed in a small tank of water filled with cold water for 6 min and sacrificed 15, 30, 90, 120 min later. (**b**) Level of hippocampal *C-fos* transcripts after subjection to cold swim measured by RT-qPCR. *$p<0.05$, ***$p\leq0.001$ determined by one-way ANOVA followed by Dunnett's post-hoc test (**c**) Level of hippocampal *Tet3* transcripts after subjection to cold swim measured by RT-qPCR. Data represent mean ± s.e.m.

Supplementary Figure S3

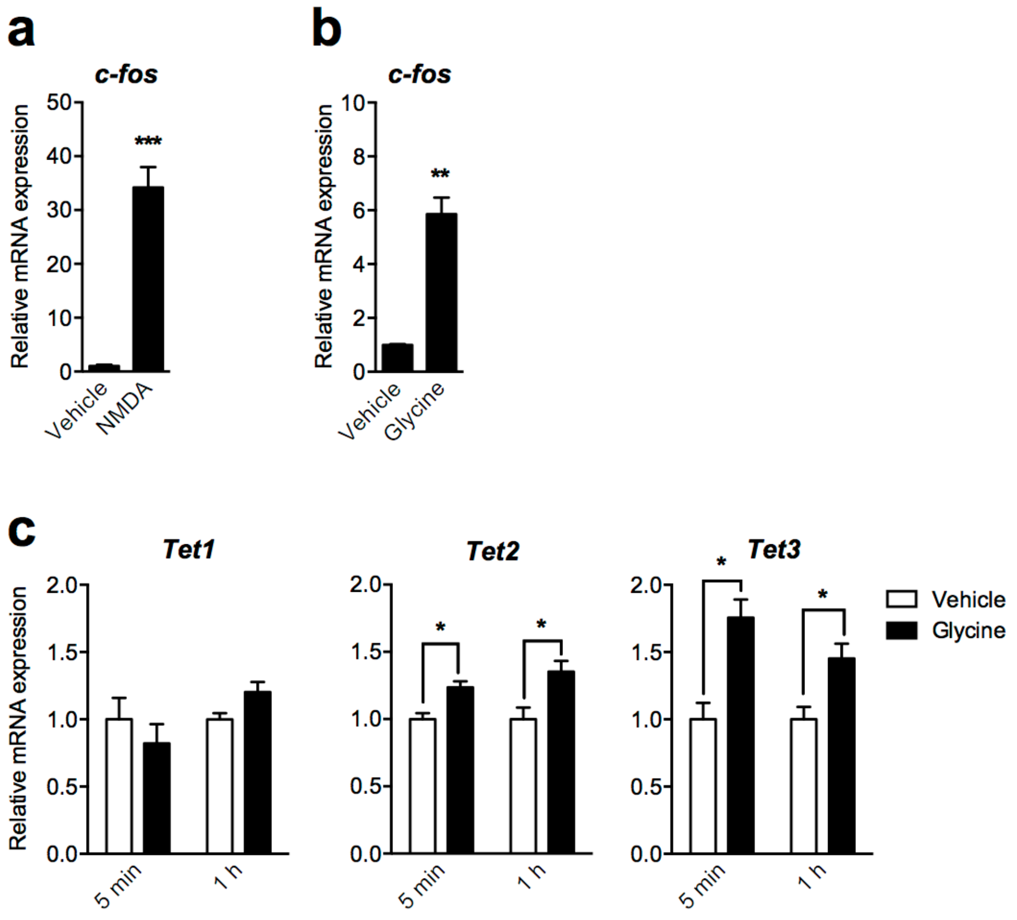

Supplementary Fig. S3. *Tet3* is responsive to activation of NMDA receptors with glycine

(**a**) Level of *C-fos* in hippocampal primary neurons 1h after NMDA stimulation measured by RT-qPCR. ****p*<0.001 determined by unpaired t test (**b**) Level of *C-fos* in hippocampal primary neurons 1h after glycine stimulation measured by RT-qPCR. **p<0.01 determined by unpaired t test (**c**) Level of *Tet1*, *2*, and *3* in hippocampal primary neurons 5 min and 1h after glycine stimulation measured by RT-qPCR. *p<0.05 determined by unpaired t test . Data represent mean ± s.e.m.

Supplementary Figure S4

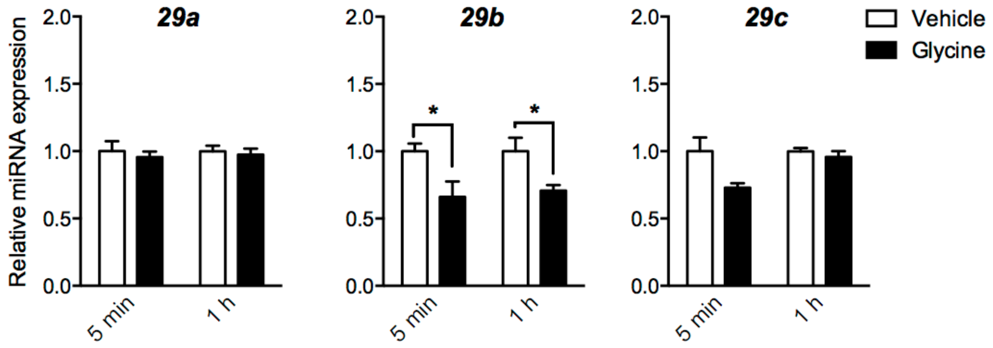

Supplementary Fig. S4. Activation of NMDA receptors with glycine leads to decreased miR-29b expression.

Level of *miR-29 a, b,* and *c* in hippocampal primary neurons 5 min and 1h after glycine stimulation measured by RT-qPCR. *p<0.05 determined by unpaired t test. Data represent mean ± s.e.m.

Supplementary Figure S5

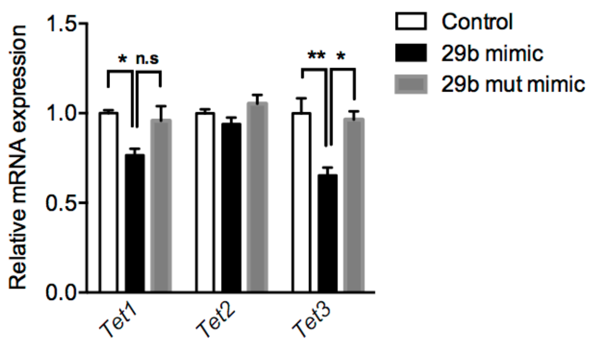

Supplementary Fig. S5. Seed mutant miR-29b mimic does not have an effect on the expression of *Tets*.

Level of *Tet1*, *Tet2,* and *Tet3* in N2a cells after transfection with miR-29b mimic, seed mutant miR-29b mimic or control measured by RT-qPCR. **p*<0.05, ***p*<0.01 determined by one-way ANOVA followed by Tukey's post-hoc test. Data represent mean ± s.e.m.

Supplementary Figure S6

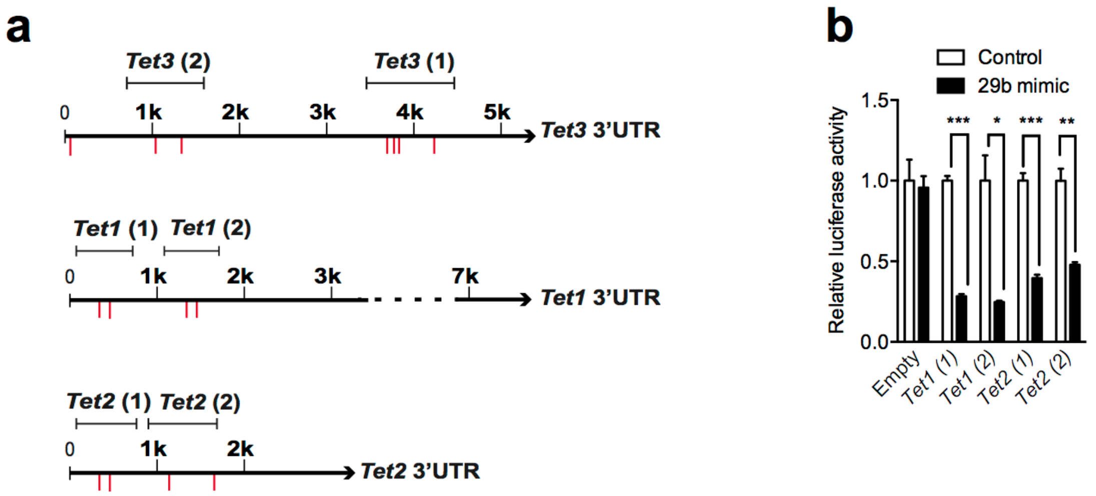

Supplementary Fig. S6. MiR-29b binds to *Tet1 and Tet2* 3' UTRs and control their expression.

(**a**) Segments of *Tet1, Tet2 and Tet3* 3'UTRs were cloned into a luciferase reporter; each segment contains conserved seed sequences (indicated in red) for the miR-29 family as predicted by TargetScan [1] (Supplementary Table S1). (**b**) Analysis of *Tet1* and *Tet2* luciferase reporters in the presence of miR-29b mimic or control (40 nM) in N2a cells. *p<0.05, **p<0.01, ***p<0.001 determined by unpaired t test. Data represent mean ± s.e.m.

Supplementary Figure S7

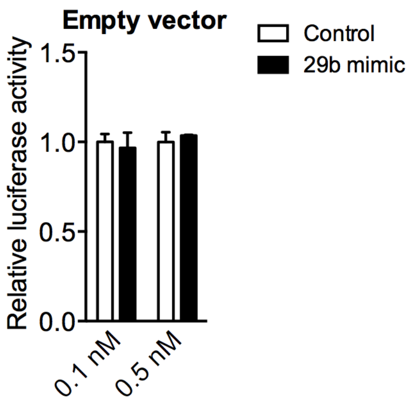

Supplementary Fig. S7. Luciferase activity of empty vector is stable in the presence of miR-29b or control.

Analysis of the luciferase activity in N2a cells transfected with empty vector in the presence of graded concentrations of miR-29b mimic or control. Data represent mean ± s.e.m.

Supplementary Figure S8

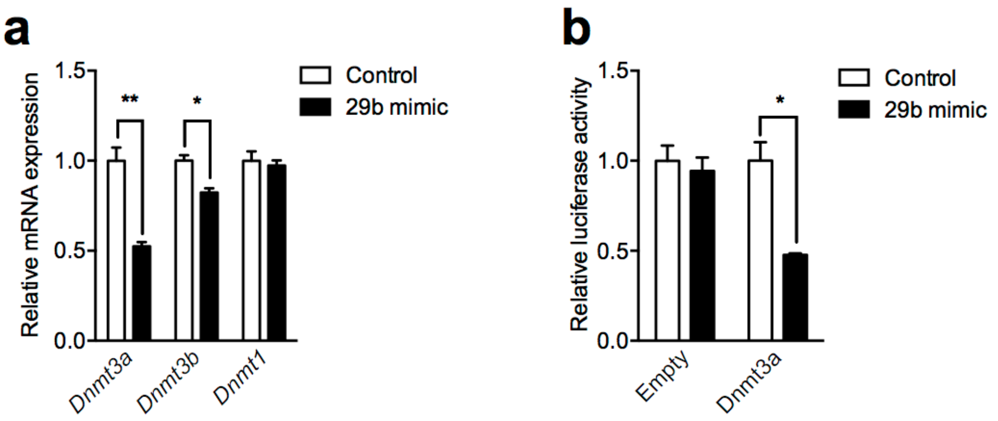

Supplementary Fig. S8. MiR-29b targets *Dnmt3a* and –*b*.

(**a**) Level of *Dnmt3a, -b* and *1* in N2a cells 24h after transfection with 29b mimic or control measured by RT-qPCR. *p<0.05, **p<0.01 determined by unpaired t test (**b**) Analysis of *Dnmt3a* luciferase reporter in the presence of miR-29b mimic or control (60 nM) in N2a cells. *p<0.05 determined by unpaired t test Data represent mean ± s.e.m.

Supplementary Figure S9

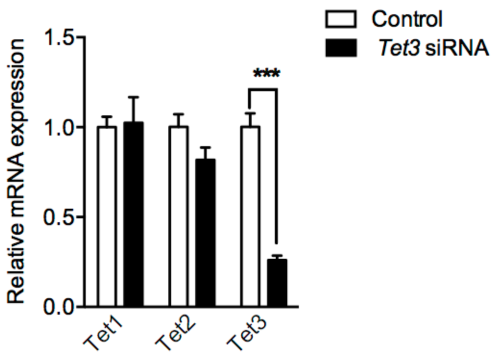

Supplementary Fig. S9. Knockdown of *Tet3* selectively reduces *Tet3* mRNA level.

Level of *Tet1*, *Tet2,* and *Tet3* in N2a cells after transfection with a pool of siRNAs directed against *Tet3* or control measured by RT-qPCR. ****p*<0.001 determined by unpaired t test. Data represent mean ± s.e.m.

Supplementary Figure S10

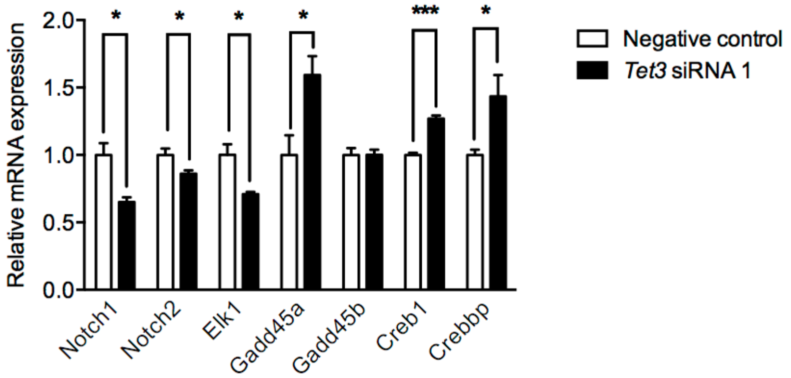

Supplementary Fig. S10. Knockdown of *Tet3* using a single siRNA alters the expression of synaptic and memory-related genes.

Transcriptional analysis of genes involved in synaptic plasticity, memory formation after *Tet3* knockdown in N2a cells by RT-qPCR. *p<0.05, ***p<0.001 determined by unpaired t test. Data represent mean ± s.e.m.

Supplementary Figure S11

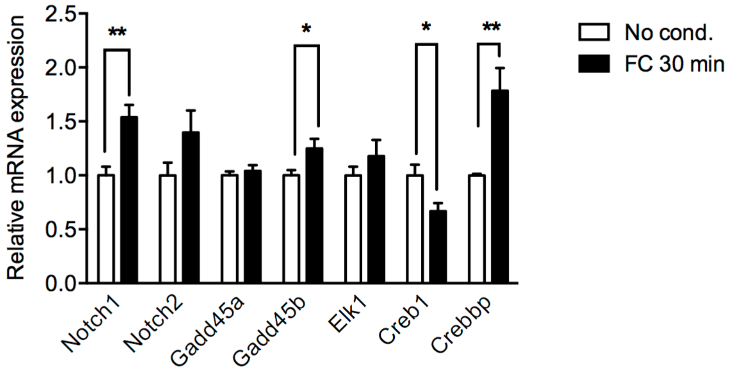

Supplementary Fig. S11. mRNA expression level of synaptic and memory-related genes in response to fear conditioning.

Level of identified *Tet3* targets in the hippocampus 30 min after fear conditioning, measured by RT-qPCR. **p*<0.05, ***p*<0.01 determined by unpaired t test. Data represent mean ± s.e.m.

Supplementary Figure S12

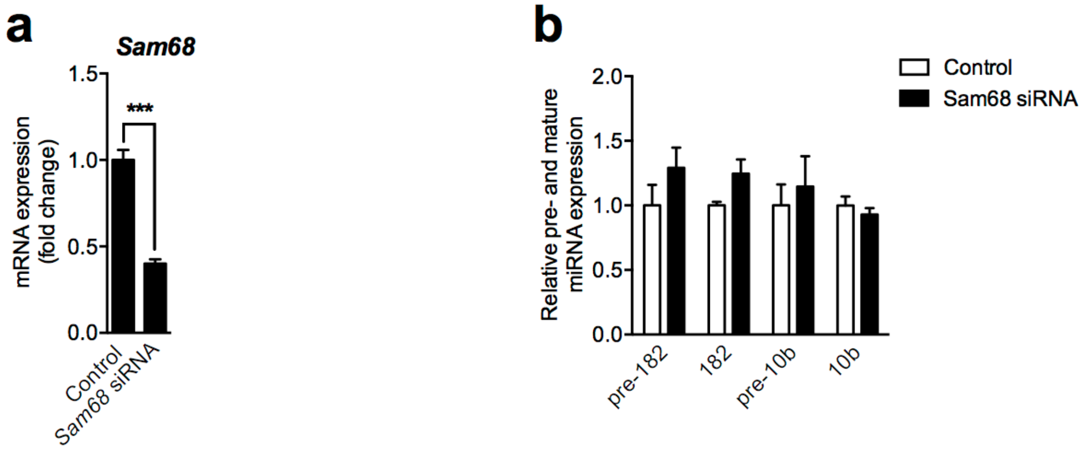

Supplementary Fig. S12. Knockdown of *Sam68* does not alter precursor and mature miR-182 and miR-10b expression level.

(**a**) Level of *Sam68* in N2a cells after transfection with a pool of siRNAs directed against *Sam68* or control measured by RT-qPCR. ****p*<0.001 determined by unpaired t test. (**b**) Level of precursor and mature miR-182 and miR-10b after transfection with a pool of siRNAs directed to *Sam68* or control measured by RT-qPCR. Data represent mean ± s.e.m.

Supplementary Figure S13

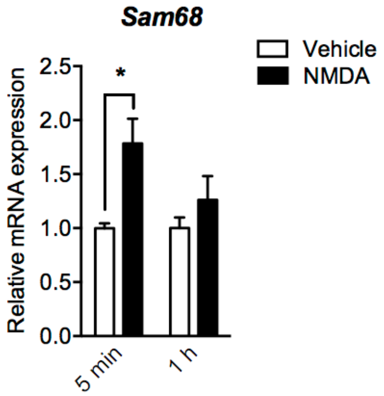

Supplementary Fig. S13. *Sam68* expression is activity-dependent. Level of *Sam68* in hippocampal primary neurons 5 min and 1 h after NMDA stimulation (60 μM, 5 min) measured by RT-qPCR. *p<0.05 determined by unpaired t test. Data represent mean ± s.e.m.

Supplementary Figure S14

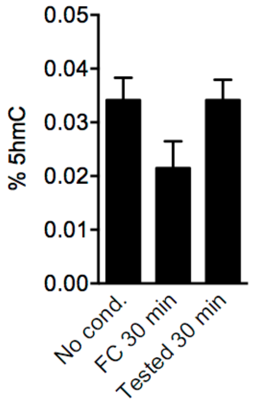

Supplementary Fig. S14. Global 5-hmC is not altered in the hippocampus after fear conditioning

Level of 5-hmC in the hippocampus 30 min after training and 30 min after testing as measured by ELISA. Data represent mean ± s.e.m.

Supplementary Figure S15

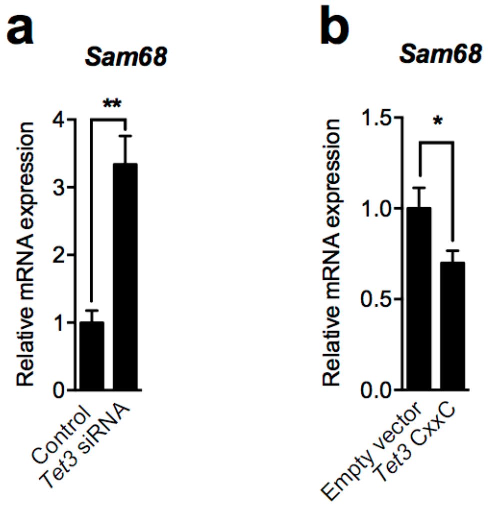

Supplementary Fig. S15. *Sam68* expression is sensitive to TET3 levels.
(**a**) Level of *Sam68* in N2a cells after transfection with a pool of siRNAs directed against *Tet3* or control measured by RT-qPCR. **p<0.01 determined by unpaired t test (**b**) Level of *Sam68* in N2a cells after transfection with a plasmid expressing *Tet3* containing the CxxC DNA-binding domain or empty vector measured by RT-qPCR. *p<0.05 determined by unpaired t test. Data represent mean ± s.e.m.

Supplementary Figure S16

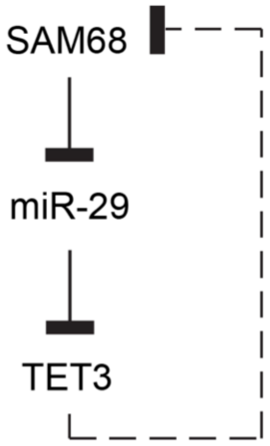

Supplementary Fig. S16. Model for the regulation of *Tet3* via miR-29b and SAM68.

SAM68 inhibits miR-29b transcription, leading to reduced mature miR-29 levels, thus allowing *Tet3* transcripts to increase. TET3 likely inhibits *Sam68* expression by binding to its promoter, thus allowing transient *Tet3* expression in response to neuronal activity.

Supplementary Figure S17

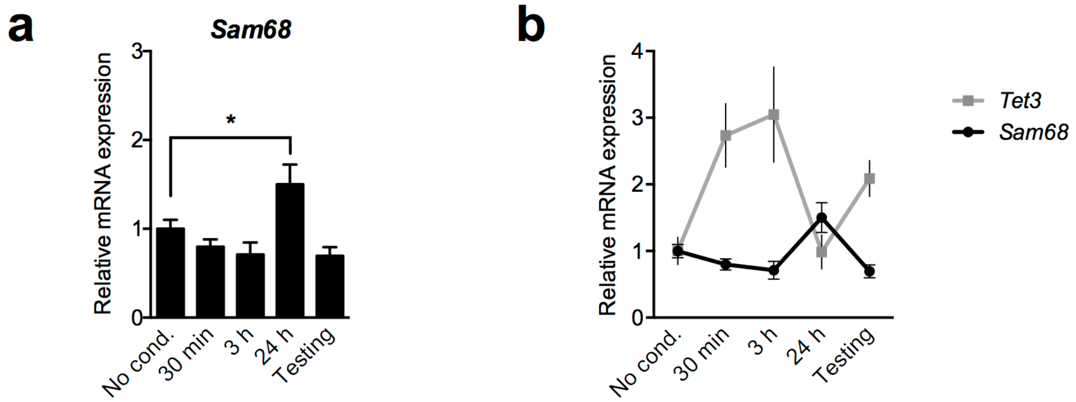

Supplementary Fig. S17. *Sam68* expression is altered in the hippocampus in response to fear conditioning.

(**a**) Level of *Sam68* in the hippocampus 30 min, 3 h, 24 h after fear conditioning, or 30 min after testing 24 h following conditioning, measured by RT-qPCR. No cond., no conditioning. *p<0.05 determined by one-way ANOVA followed by Dunnett's post-hoc test. (**b**) Superposition of the data depicted in Fig. 1a and Fig. S19a. *Tet3* and *Sam68* levels are negatively correlated, suggesting the presence of a negative feedback loop. It should be noted samples were collected at consistent time points and do not permit to resolve a delay in this putative feedback reaction, which would be expected if TET3 proteins regulate *Sam68* expression at the transcriptional level.

SUPPLEMENTARY TABLES

3'UTR	Site position	Seed position	Seed type	P_{CT}
Tet3	42-57	51-57	7mer-m8	0.83
	1032-1039	1032-1038	7mer-m8	0.82
	1358-1373	1367-1373	7mer-m8	0.73
	3680-3706	3699-3705	8mer	0.89
	3721-3753	3747-3752	7mer-A1	0.87
	3807-3831	3824-3830	7mer-m8	0.73
	4248-4265	4258-4264	8mer	0.85
Tet1	297-322	316-322	7mer-m8	0.68
	407-423	417-423	7mer-m8	0.89
	1326-1359	1353-1358	7mer-A1	0.7
	1399-1430	1424-1430	7mer-m8	0.89
Tet2	367-405	398-404	7mer-m8	0.68
	525-564	557-563	8mer	0.89
	1032-1061	1055-1061	7mer-m8	0.7
	1587-1621	1614-1620	8mer	0.89

Supplementary Table S1. Modified output table of TargetScanMouse analysis.

List of complementary sites for miR-29s in the 3'UTR region of *Tet1*, *2* and *3* predicted by TargetScanMouse [1]. *Tet3* 3'UTR contains seven well-conserved miR-29s binding sites, while *Tet1* and *Tet2* has four only. The site position represents the distance (bp) between the stop codon and binding sites of miR-29b. The seed position represents the distance (bp) between the stop codon and binding sites of the seed sequence of miR-29b. P_{CT} is defined as the probability of conserved targeting [2].

mmu-miR-29b-3p	Site position	Seed position	ΔG$_{hybrid}$	Site Access	Seed Access
Tet3 3'UTR	42-57	51-57	-22	0.35	0.447
	963-994	989-994	-18.8	0.365	0.333
	982-1005	1000-1005	-19.5	0.33	0.202
	1010-1016	1010-1015	-16.7	0.294	0.325
	1013-1026	1019-1025	-22.6	0.262	0.17
	1032-1039	1032-1038	-17.5	0.198	0.225
	1148-1164	1159-1164	-21.3	0.477	0.291
	1310-1336	1331-1336	-18.8	0.476	0.314
	1358-1373	1367-1373	-18.4	0.391	0.386
	3062-3082	3077-3082	-21.4	0.455	0.447
	3327-3338	3332-3337	-16.5	0.481	0.291
	3680-3706	3699-3705	-22.9	0.509	0.344
	3721-3753	3747-3752	-20.5	0.58	0.365
	3807-3831	3824-3830	-19.7	0.301	0.286
	4069-4086	4080-4085	-19.3	0.447	0.47
	4178-4201	4195-4201	-20.6	0.452	0.432
	4205-4220	4215-4220	-15.6	0.311	0.406
	4248-4265	4258-4264	-23.1	0.278	0.357
Tet1 3'UTR	6454-6490	6483-6489	-25.7	0.346	0.458
	407-423	417-423	-20.4	0.306	0.409
	79-96	90-95	-18.3	0.372	0.241
	2043-2082	2076-2082	-19.1	0.338	0.253
	297-322	316-322	-18.1	0.453	0.555
	1326-1359	1353-1358	-21.5	0.304	0.49
	1521-1537	1532-1537	-19	0.44	0.178
	1399-1430	1424-1430	-21.7	0.412	0.854
	5250-5275	5269-5275	-22.5	0.464	0.446
	6987-7004	6999-7004	-20.6	0.316	0.18
	4551-4581	4574-4580	-24.5	0.241	0.041
	457-474	469-474	-20.5	0.193	0.204
	7651-7665	7660-7665	-16.2	0.154	0.032
	1455-1476	1471-1476	-17.9	0.305	0.144
Tet2 3'UTR	1587-1621	1614-1620	-23.3	0.375	0.13
	525-564	557-563	-28.6	0.417	0.463
	1032-1061	1055-1061	-19	0.465	0.456
	367-405	398-404	-25	0.203	0.268
	1129-1168	1161-1167	-20.8	0.333	0.07

Supplementary Table S2. Modified output table of STarMiR analysis.

Free energy in kcal/mol (ΔGhybrid) analysis of putative miR-29b binding site to each *Tet* 3'UTR, and measure of the structural accessibility in the predicted binding site (Site Access) or in the target sub-region complementary to the miRNA seed (Seed Access) as defined by STarMir [3]. Highlighted rows are conserved miR-29b binding sites as predicted by TargetScanMouse [1] (S1 Table).

Genes	Forward primer (5'->3')	Reverse primer (5'->3')
Tubd1	TCTCTTGCTAACTTGGTGGTCCTC	GCTGGGTCTTTAAATCCCTCTACG
Hprt1	GTTGGGCTTACCTCACTGCTTTC	CCTGGTTCATCATCGCTAATCACG
Actb	CAACGGCTCCGGCATGTGC	CTCTTGCTCTGGGCCTCG
Gapdh	CAGCAATGCATCCTGCACC	TGGACTGTGGTCATGAGCCC
Tet1	TTGCTGGAGACTGTCGACTTGG	TGCTCGAATCAACGTACACACCAC
Tet2	TGCCAAATGGCAGTACAGTGGTG	ATCCTCAGGCTTAGCTCCGACTTC
Tet3	GCATCGGGCAGGCCACCATT	GGCAAGCACAGGTCCGGTCA
Dnmt3a	CAGCTGCTTACGCCCCACCC	CACCAGCCGCTCCCTTGTGC
Dnmt3b	AAAGCCCGGCTGTCCGAACC	CCCTGCCGACCTCGGGTGAT
Dnmt1	AGTCTGTTCCTGTGCAGAAGGC	TGCTGAAGAAGCCATCCCACTC
Fos	ACAGATACACTCCAAGCGGAGAC	TGGCAATCTCAGTCTGCAACGC
Drosha	CATCACGAAGGACACTTGACGTTG	TGCTACCTTGGCTTGCGTTCTG
Dgcr8	GTCACTTGGTCCAGACCCTACTTC	GCTTAGAGGAGGATCATGTTTCCG
Dicer	TCTTCGAG CTCCATTGTTGGTC	CTACCACTCTTTCACCAACCG
Ddx5	ACCATTGACGCCATGTCGAG	CAAATCGAGGTGCACCAAACCC
Ddx17	AGGGATATGGTTGGCATTGCACAG	CAATCGCAGGCAGCAAATACGC
p53	CACGTACTCTCCTCCCCTCAAT	AACTGCACAGGGCACGTCTT
Notch 1	ACAGTGCAACCCCCTGTATG	TCTAGGCCATCCCACTCACA
Notch 2	ACAGTGTTGGCTCCCTGTTC	ATCGTTTACCTTGCCAGCCA
Khdrbs1	TTATGGCCCATGCTATGGAAGA	AGGTACTCCGTTCAAGTAGGAC
Elk1	CTGCTCCCCACACATACCTT	GAGAGGCCATCCACACTGAT
Elk4	ATCTAACAATGGGGAGTTCAAGC	GGCTCGGCTGAGTTTATCATAAT
Gadd45a	TGC GAG AAC GAC ATC AAC AT	TCC CGG CAA AAA CAA ATA AG
Gadd45b	CTGCCTCCTGGTCACGAA	TTGCCTCTGCTCTCTTCACA
Tdg	TAGGAAACGTGCGTGTTCAG	CTCATACTGCCAAACCAGCA
Crebbp	TGGAGTGAACCCCCAGTTAG	TTGCTTGCTCTCGTCTCTGA
Creb1	AGCTGCCACTCAGCCGGGTA	TCGCCTGAGGCAGCTTGAACA
**miRNAs**	**Source**	
miR-29b-1	Qiagen, cat. No.: MS00005936	
miR-29a	Qiagen, cat. No.: MS00001372	
miR-29c	Qiagen, cat. No.: MS00001379	
miR-10b	Qiagen, cat. No.: MS00032249	
miR-182	Qiagen, cat. No.: MS00011291	
Snord61_11	Qiagen, cat. No.:MS00033705	
Rnu6	Qiagen, cat. No.: MS00033740	
Pre-miR-29b-1	Qiagen, cat. No.: MP00005355	
Pre-miR-29a	Qiagen, cat. No.: MP00005348	
Pre-miR-29c	Qiagen, cat. No.: MP00005369	
Pre-miR-10b	Qiagen, cat. No.: MP00003983	
Pre-miR-182	Qiagen, cat. No.: MP00004431	
Pri-miR-29a/b-1	AACTATTGCACGGACTTCACCT	TCCTGA AGA AGCTTTGTCGTC

Supplementary Table S3. List or primers used for the quantification of mRNA and miRNA transcripts.

**Name**	**Forward primer (5’->3’)**	**Reverse primer (5’->3’)**
Tet1 (1)	AATGCCTTTGCTAATGTGGTG	TTAGCGAACAGCTTCCAACC
Tet1 (2)	AGGAAAATGGGAACCCAAAC	TGAGGGAGGATTTCTGATGG
Tet2 (1)	AATGCCTTTGCTAATGTGGTG	TTAGCGAACAGCTTCCAACC
Tet2 (2)	TCGGCTGATGAGCAGTATCA	AGCAATCTGGGTAGCACCAT
Tet3 (1)	TTTAAAGAAACAGTAGTTTGCAGAGC	TATCATACCCTCATGGAATCTAAGTT
Tet3 (2)	GCTCTTCTCGTCCCGTTGAT	TAGAGCCACGTGCTAACTGC
Dnmt3a	TTGGCCTTGCAAAAGGGTTG	TTGCACGCGAGTCTGGATAA

Supplementary Table S4. List or primers used for cloning

Supplementary Table S5. Numerical data and statistical analysis used in all figures.

References

1 Lewis, B. P., Burge, C. B. & Bartel, D. P. Conserved seed pairing, often flanked by adenosines, indicates that thousands of human genes are microRNA targets. *Cell* **120**, 15-20, doi:10.1016/j.cell.2004.12.035 (2005).

2 Friedman, R. C., Farh, K. K., Burge, C. B. & Bartel, D. P. Most mammalian mRNAs are conserved targets of microRNAs. *Genome research* **19**, 92-105, doi:10.1101/gr.082701.108 (2009).

3 Ding, Y., Chan, C. Y. & Lawrence, C. E. Sfold web server for statistical folding and rational design of nucleic acids. *Nucleic Acids Res* **32**, 135-141, doi:10.1093/nar/gkh449 (2004).
